# Readability of patient education materials related to radiation safety: What are the implications for patient-centred radiology care?

**DOI:** 10.1186/s13244-021-01094-3

**Published:** 2021-10-21

**Authors:** Francis T. Delaney, Tiarnán Ó. Doinn, James M. Broderick, Emma Stanley

**Affiliations:** 1 Department of Radiology, Mater Misericordiae University Hospital, Dublin, Ireland; 2 Department of Trauma and Orthopaedic Surgery, Tallaght University Hospital, Dublin, Ireland

**Keywords:** Patient-centred care, Health literacy, Radiation dosage, Patient education, Readability

## Abstract

**Background:**

Increasing numbers of patients and carers rely on online resources for healthcare information. Radiation safety can be misunderstood by patients and clinicians and lead to patient anxiety. We aimed to assess the readability of online patient educational materials (PEMs) related to radiation safety.

**Methods:**

A total of 84 articles pertaining to radiation safety from 14 well-known online resources were identified. PEMs were then analysed using Readability Studio Professional Edition Version 2019. Readability was assessed using eight different instruments: the Flesch-Kincaid Reading Grade Level, Raygor Estimate, SMOG, Coleman–Liau, Fry, FORCAST, Gunning Fog, and Flesch Reading Ease Score formula. The mean reading grade level (RGL) of each article was compared to the 6th and 8th grade reading level using 1-sample t-tests.

**Results:**

The cumulative mean RGL for all 84 articles was 13.3 (range = 8.6–17.4), and none were written at or below the 6th or 8th grade level. The cumulative mean RGL exceeded the 6th grade reading level by an average of 7.3 levels (95% CI, 6.8–7.8; *p* < 0.001) and the 8th grade level by an average of 5.3 grade levels (95% CI, 4.8–5.8; *p* < 0.001). The mean Flesch Reading Ease Score was 39/100 (‘difficult’).

**Conclusion:**

Currently available online PEMs related to radiation safety are still written at higher than recommended reading levels. Radiation safety is a topic in which the specialist training of radiologists is crucial in providing guidance to patients. Addressing the readability of online PEMs can improve radiology-patient communication and support the shift to a patient-centred model of practice.

**Supplementary Information:**

The online version contains supplementary material available at 10.1186/s13244-021-01094-3.

## Key points

- Patients increasingly use the internet to access healthcare information on which medical decisions are based.
- Radiation safety is a complex topic which can cause uncertainty among patients and clinicians making specialist knowledge from radiologists important in guiding clinical practice.
- Online patient education material is often written at a higher than recommended reading level which is a limitation to informed decision-making in a model of patient-centred care.

## Background

Internet usage is increasing worldwide with 87% of adults and 98% of young adults in developed countries now regularly online [1]. Widespread internet access has transformed how people obtain medical information. Up to 85% of internet users access healthcare information online and for 70% the internet is their primary resource for medical questions [2, 3]. There is substantial demand for online information related to radiology with an online patient resource RadiologyInfo.org averaging nearly 1 million monthly visits [4]. Half of those who access healthcare information online report that it influences their decision making [5]. However, medical information obtained through standard internet searches can be misleading for patients [6].

The paradigm shift in the physician–patient relationship as patients can now readily access medical information independently online has generated a greater focus on improving health literacy. Health literacy is a person’s ability to access, read and understand medical information and make informed decisions [7, 8]. In the United States (US), only 12% of adults have proficient health literacy, while 36% have basic or below basic levels of health literacy, and findings in other western countries are similar [9, 10]. Limited health literacy is associated with lower quality of life, higher medical costs, increased hospitalisations, and worse outcomes including increased mortality [7, 11, 12]. Moreover, it has a significant economic impact and is estimated to cost up to 236 billion dollars annually in the USA [11, 12].

Readability, “the determination by systematic formulae of the reading comprehension level a person must have to understand written materials”, correlates with literacy and is a major factor in determining whether a patient can understand medical information [8, 13]. The US reading grade level (RGL) denotes the years of education required to easily read and understand a piece of text. The average American reads at a 7th–8th grade level and 20% read at a 5th grade level or below [7, 8, 14]. Therefore, organisations such as the American Medical Association (AMA), the National Institutes of Health (NIH), and the Agency for Healthcare Research and Quality (AHRQ) recommend that patient education materials (PEMs) be written at or below a 6th grade reading level [7, 14, 15]. However, studies have consistently demonstrated that online PEMs are produced at a higher RGL than recommended. In a study of online PEMs across 16 medical specialities, none met the average adult reading ability and diagnostic radiology was among the specialities with the highest RGL [16]. PEMs which are difficult to understand may encourage patients to seek information from simpler but less reliable online resources [6].

Concern regarding radiation exposure has grown in recent decades as the volume of imaging tests performed increased, with radiology examinations accounting for half of all radiation exposure to the US population by 2009 [17]. However, there is a general lack of understanding of the risks of radiation exposure with a systematic review in 2010 reporting that “only a minority of physicians were well-informed” about radiation doses and radiation risks and that less than a quarter discussed risks with their patients [18]. Two-thirds of people report worrying about the health risks associated with radiation exposure during imaging tests, and 12% report high levels of worry [19]. Patient anxiety may negatively impact quality of life and lead to avoidance of imaging tests [19].

A 2013 study by Hansberry et al. analysed the readability of PEMs related to radiation safety from eight online resources [20]. PEMs from all websites were written well above the recommended RGL, and only 3 of 45 articles were written below a 10th grade level. Similarly, Yi et al. in 2016 showed that online PEMs related to paediatric radiation safety were also written above recommended RGLs [21]. Therefore, the goal of this study was to assess the readability of currently available online PEMs related to radiation safety and determine whether there has been any improvement since 2013.

## Materials and methods

Using a cross-sectional study design, fourteen online resources with education material related to radiation exposure and radiation safety in medical imaging were identified in December 2020 based upon previous studies, internet searches, and author experience (Table 1). Resources were divided into academic or non-profit organisations during analysis (Table 1). Academic resources were those affiliated with a university, academic medical centre or medical society, and non-profit were those operated by government or non-profit organisations. Articles written in English with sufficient text to analyse were included, and a total of 84 articles were analysed.

**Table 1:** Web-based patient education materials by website and mean reading grade

	Website	Number of articles (%)	Mean reading grade (range)
Academic	Image gently	6 (20.7%)	12.9 (8.8–14.4)
	ESR	6 (10.9%)	13.4 (10.9–16.0)
	Inside radiology	3 (10.3%)	12.4 (11–14.2)
	Mayo Clinic	4 (13.8%)	11.3 (9.6–10.1)
	Radiology info	13 (44.8%)	12.9 (10.8–17)
	Society for Paediatric Radiology	3 (10.3%)	13.2 (10.8–15.1)
Non-Profit	CDC	7 (12.7%)	12.6 (10.2–13.8)
	EPA	6 (10.9%)	13.4 (11.4–15)
	FDA	12(21.8%)	14.9 (11–17.2)
	Health Physics Society	4 (7.2%)	15.4 (14.5–15.9)
	International Atomic Energy Agency	6 (10.9%)	13.2 (11.9–15.4)
	Medline Plus	3 (5.5%)	10.2 (8.8–12.3)
	Nuclear Regulatory Commission	8 (14.5%)	15.4 (13.8–17.4)
	Patient info	3 (5.5%)	8.7 (8.6–8.8)
Total		84 (100%)	13.3 (8.6–17.4)

ESR, European Society of Radiology; CDC, Centers for Disease Control and Prevention; EPA, Environmental Protection Agency; FDA, U.S. Food & Drug Administration

Article text was copied into separate Microsoft Word documents (Microsoft, Redmond, WA). Text not related to educational material such as hyperlinks, photographs or advertisements was removed. The reformatted patient education resources were then analysed using Readability Studio Professional Edition Version 2019, Oleander Software Ltd. [22]. Readability was assessed using eight different instruments (Table 2) which are widely used in medical literature and described on the Readability Studio platform [20–24]. Seven of these analysed the RGL including the Flesch–Kincaid Reading Grade Level (FKGL), Raygor Estimate, SMOG, Coleman–Liau, Fry, FORCAST and Gunning Fog [22]. RGLs were reported as a US grade level and for each word document the seven RGL tests generated seven RGL scores, as well as a mean RGL. Unlike the other readability formulae, the Flesch Reading Ease Score (FRES) formula calculates the readability based on sentence length and number of syllables, expressed as an index score from 0–100 [22]. Scores of 0–30 indicate ‘very difficult’, 30–50 are ‘difficult’, 50–60 are ‘fairly difficult’, 60–70 are ‘standard’, 70–80 are ‘fairly easy’, 80–90 are ‘easy’, and 90–100 are ‘very easy’.

**Table 2:** Summary of readability formulae

Readability test	Score Type	Description	Formula
Flesch–Kincaid reading grade level	Grade level	Part of the Kincaid Navy Personnel collection of tests. Designed for technical documents and suited to a broad array of disciplines	*G* = (11.8 × (*B*/*W*)) + (0.39 × (*W*/*S*)) − 15.59
Flesch–Kincaid reading ease	Index score (0–100)	Developed to assess the readability of newspapers. Best suited to assessing school textbooks and technical manuals. Standard test used by many US government agencies. Scores range from 0–100, with higher scores denoting easier readability	*I* = (206.835 − (84.6 × (*B*/*W*)) − (1.015 × (*W*/*S*)))
The Raygor estimate	Grade Level	Designed for most text, including literature and technical documents	Calculated using the mean number of sentences and long words (≥ 6 characters) per 100 words, which are plotted on to a RE Graph, where their intersection determines RGL
Fry	Grade level	Designed for a variety of texts including technical documents and literature, across a range of levels, from primary school level to university level	Calculated using the mean number of sentences and syllables per 100 words, which are plotted on to a Fry Graph, where their intersection determines RGL
SMOG	Grade level	Generally appropriate for secondary age (4th grade to college level) readers. Tests for 100% comprehension, whereas most formulas test for around 50–75% comprehension. Most accurate when applied to documents ≥ 30 sentences in length	*G* = 1.0430 × √*C* + 3.1291
Coleman–Liau	Grade level	Designed for secondary age (4th grade to college level) readers. Formula is based on text from the .4 to 16.3 grade level range. Applicable to numerous sectors	*G* = (− 27.4004 × (*E*/100)) + 23.06395
FORCAST	Grade level	Devised for assessing U.S. Army technical manuals and forms. It is the only test not designed for running narrative	*G* = 20 − (M/10)
Gunning fog	Grade level	Developed to assist American businesses improve the readability of their writing. Applicable to numerous disciplines	*G* = 0.4 × (*W*/*S* + ((C*/*W*) × 100))

*G*, grade level; *B*, number of syllables; *W*, number of words; *S*, number of sentences; RGL, Reading Grade Level; *I*, Flesch Index Score; RE, Raygor Estimate; SMOG, Simple Measure of Gobbledygook; *C*, complex words (≥3 syllables); *E* , predicted Cloze percentage = 141.8401 − (0.214590 × number of characters) + (1.079812*S); *M*, number of monosyllabic words; C*, complex words with exceptions including, proper nouns, words made 3 syllables by addition of "ed" or "es", compound words made of simpler words

Data were also provided on the number and percentage of complex words, long words, Dale-Chall unfamiliar words, as well as the number of 'wordy' items, overly long sentences and longest sentence length [22]. Complex words are defined as words with ≥ 3 syllables and long words as those with ≥ 6 characters. Dale-Chall unfamiliar words are defined as those that do not appear on a list of 3000 common words that are known to most 4th-grade students. 'Wordy' items include complex words and phrases that contain too many words. Overly long sentences are defined as those with a word count greater than 22 words.

The number of articles with a RGL less than or equal to the 8th grade (average US adult reading level) and 6th grade (recommended level for PEMs) was determined. The mean RGL of each article was compared with the 6th grade and 8th grade reading levels using 1-sample t-tests. The mean RGL of academic and non-profit website’s articles was compared using an independent t-test. The mean percentage of linguistic units was also compared using a one-way analysis of variance (ANOVA). Post hoc analysis was performed using Games-Howell tests. All statistical analysis was carried out in IBM SPSS Statistics for Windows, version 26 (IBM Corp., Armonk, N.Y., USA).

## Results

**Fig. 1:**
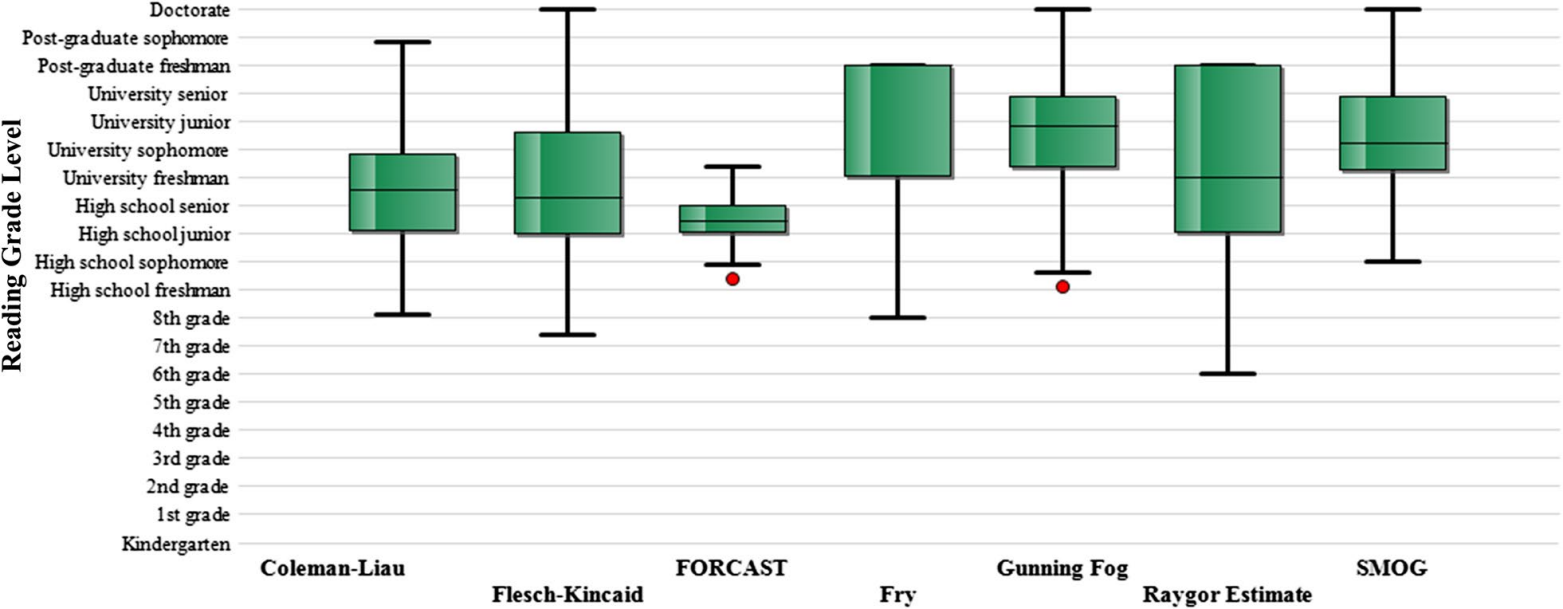
Summary of the mean reading grade level for each readability test

**Fig. 2:**
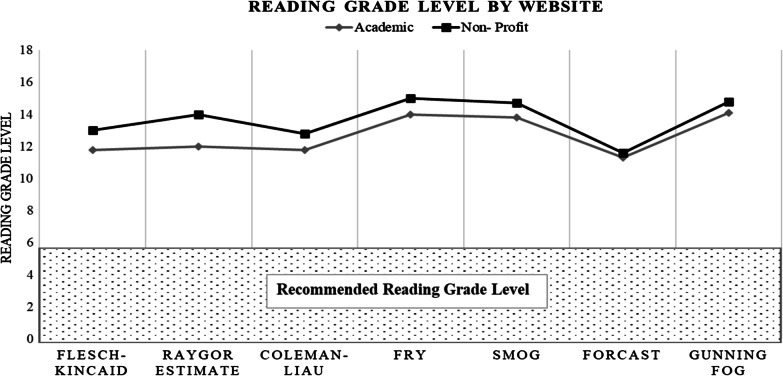
Mean reading grade level for each website

Fourteen websites were included with a total of 84 education materials related to radiation exposure and safety. These included the eight websites included in the study by Hansberry et al. and four analysed by Yi et al. [20, 21]. There were 6 academic (*n* = 35) and 8 non-profit (*n* = 49) websites (Table 1). The cumulative mean RGL for all 84 articles was 13.3 (range = 8.6–17.4) (Fig. 1 and Table 1). There was no significant difference (*p* = 0.052, 95% CI − 1.9 to 0.08) between the cumulative mean RGL of academic websites (mean = 12.7, SD ± 1.9) and non-profit (mean = 13.6 SD ± 2.3) websites (Table 3 and Fig. 2). Examining the mean RGL for each article revealed that no article (0%) was written at or below the 6th or 8th grade reading level. The cumulative mean RGL of the articles exceeded the 6th grade level by an average of 7.3 grade levels (95% CI, 6.8–7.8; *p* < 0.001) and the 8th grade level by an average of 5.3 grade levels (95% CI, 4.8–5.8; *p* < 0.001). Of the 84 PEMs analysed, 10 (11.9%) could not be evaluated via the Fry test and 8 (9.5%) could not be evaluated via the Raygor Estimate due to too many complex words. The mean FRES index was 39 which is classified as ‘difficult’.

**Table 3:** Mean reading grade level of patient education articles

Test	Academic (range)	Non-profit (range)	*p* value
Flesch–Kincaid	11.8 (8–17.3)	13.0 (7.4–19)	
Raygor estimate	12 (7–17)	14 (6–17)	
Coleman–Liau	11.8 (8.5–17.8)	12.8 (8.1–17.8)	
Fry	14 (8–17)	15 (8–17)	
SMOG	13.8 (10.5–18.4)	14.7 (10.0–19.0)	
FORCAST	11.3 (9.9–12.8)	11.6 (9.4–13.4)	
Gunning fog	14.1 (9.7–19.0)	14.8 (9.1–19)	
Mean	12.7 (8.8–17.0)	13.6 (8.6–17.4)	*p* = 0.052

**Fig. 3:**
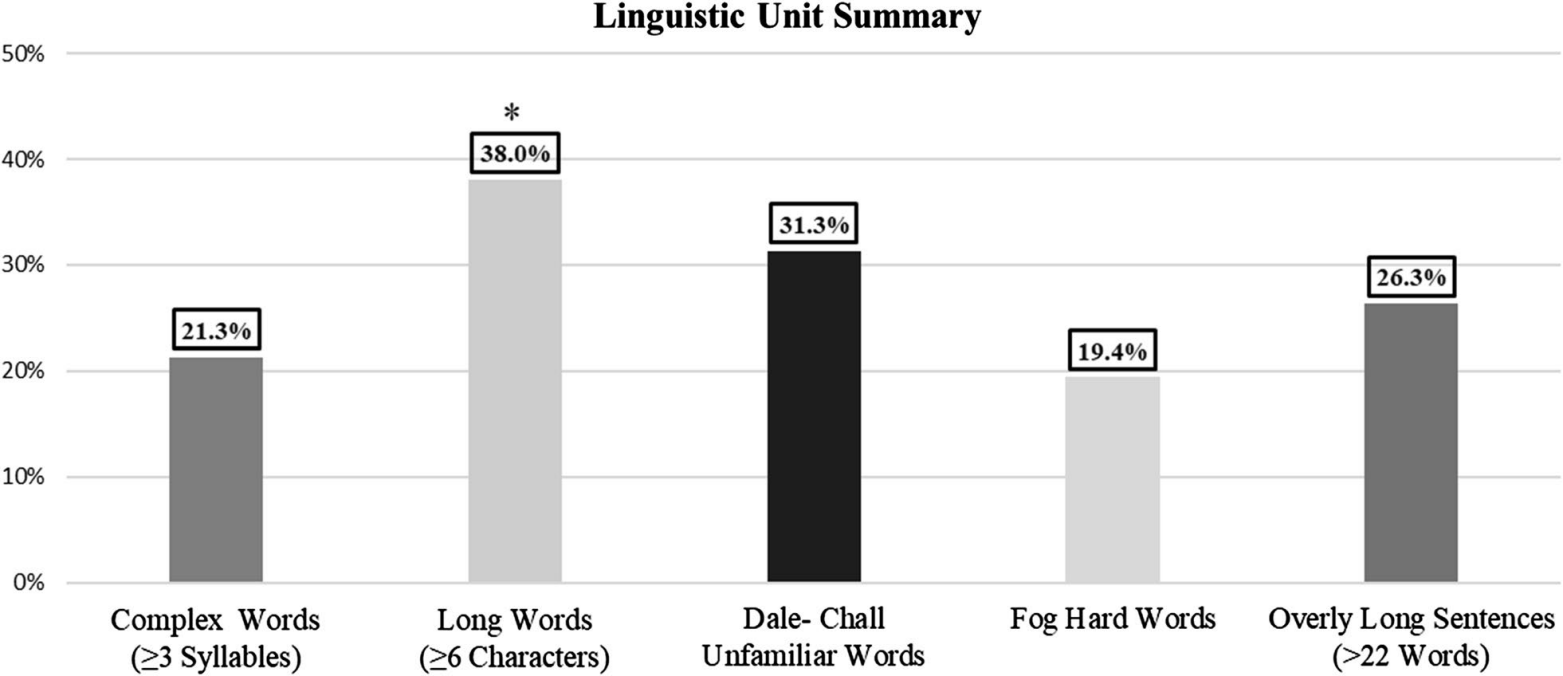
Summary of key linguistic units

A summary of key linguistic units across all articles is presented in Fig. 3. There was a significantly higher mean percentage of long words (mean = 38.0, SD ± 5.6) compared to complex words (*p* < 0.001, 95% CI, 14.2–19.1), Fog hard words (*p* < 0.001, 95% CI, 16.2–20.8), Dale-Chall unfamiliar words (*p* < 0.001, 95% CI, 3.8–9.5), and overly long sentences (*p* < 0.001, 95% CI, 7.1–16.2). The mean longest sentence across all articles was 41.7 words (range = 25–110). Table 4 lists fifty common ‘wordy’ items with suggested alternatives. As a reference, all ‘wordy’ items and suggested alternatives are listed alphabetically in Additional file 1: Appendix 1. A list of the titles of the articles included from each website is supplied in Additional file 1: Appendix 2.

**Table 4:** Selection of ‘wordy’ items with suggested alternatives produced by readability studio software (‘wordy’ items are complex words and phrases that contain too many words)

Wordy item	Suggested alternative
Abbreviated	Shortened
Accuracy	Correctness, exactness
Acquire	Gain, get
Adequate	Enough
Adverse	Harmful
Component	Part
Conscious	Aware
Consequences	Results
Criteria	Requirements
Deliberately	On purpose
Demonstrate	Show
Detrimental	Harmful
Distinguish	Tell apart
Equivalent	Equal
Evaluate	Check, rate
Facilitate	Ease, help
Feasible	Can be done
Hazardous	Risky, unsafe
Implement	Carry out, do
In reference to	About, on, for, as for, in, of, over, respecting, to, toward, with
In the process of	While
In the vicinity of	Close to, near, about, close by, in, nearby, around, round, close
Magnitude	size
Modify	Change
Necessitated	Caused, needed
Numerous	Many
Optimum	Best, greatest
Pertaining to	About, regarding
Proceed	Do, go on
Radiant	Bright
Recommend	Suggest
Regulation	Rule, law
Requisite	Needed, necessity
Stringent	Strict, tight
Sufficient	Enough, ample
Supplemental	Added, extra

## Discussion

The shift towards patient-centred care and a model of partnership between patients and providers in which patients are the primary decision-makers is an important ongoing development in healthcare practice [11, 25]. One of the key steps in achieving an effective system of shared decision-making is adequate provision of information to patients [26]. There is a focus on improving communication between radiologists and patients in recognition of this [27]. However, opportunities for direct radiologist-patient contact are limited and high-quality online PEMs are a potential means to explain important topics such as radiation safety to patients [25]. Unfortunately, as demonstrated here and in other studies, the readability of online PEMs continues to exceed recommended levels [28, 29].

None of the 84 articles were written at or below either the recommended RGL (6th grade) or the average US adult reading level (8th grade). The cumulative mean RGL was 13.3, more than double the recommended level. Patient Info, a UK-based and NHS supported website, had the lowest RGL of 8.7, though only three articles were included as it is a general healthcare site with limited material dedicated to radiology. The resources with the highest mean RGLs were the Health Physics Society and the Nuclear Regulatory Commission both of which had a cumulative mean RGL of 15.4. These three resources are from non-profit organisations. Among the academic organisations, the cumulative mean RGL ranged from 11.3 (Mayo Clinic) to 13.4 (European Society of Radiology—ESR). There was no statistically significant difference between the mean RGLs of the academic versus the non-profit organisation websites (*p* = 0.052) and the most appropriate resource to recommend for patients beyond the limited information on Patient Info remains unclear.

In comparing our results to those of Hansberry et al., there has not been a significant improvement in the readability of online PEMs related to radiation safety since 2013 [20]. While Patient Info was not analysed in the previous study, its easier readability is encouraging. Medline Plus was among the sites with the lowest RGL in 2013 (at 11.5) and has improved further with a current cumulative mean RGL of 10.2. Of the other resources analysed in 2013, the Mayo Clinic, Centers of Disease Control, Environmental Protection Agency, ESR, Food and Drug Administration, and Nuclear Regulatory Commission show limited change in our analysis with no variation in RGL of greater than 0.4. An increase in mean RGL of materials from the Society for Paediatric Radiology (SPR) website likely reflects the creation of Image Gently, but it is noteworthy that materials from this recently created resource are also written at a higher than recommended level with a cumulative mean RGL of 12.4. However, more encouragingly and in response to studies analysing the readability of online PEMs, RadiologyInfo.org has committed to improving its readability through measures such as addition of non-prose teaching aids and feedback surveys from users [30].

The use of imaging across healthcare continues to increase, and decisions regarding radiology examinations have a key role to play in an effective system of patient-centred care [17]. Radiation safety is a complex topic with the radiation exposure from different imaging tests and our understanding of the resultant risk constantly evolving. In fact, the dose from many examinations is dropping with the average dose from individual imaging tests decreasing by 15–20% between 2006 and 2016 [17]. Determining the actual risk from a particular imaging test is challenging, and the risk for most patients is outweighed by the potential benefits. Large exposures to radiation, such as nuclear accidents, clearly carry a high risk of developing cancer, but the risk associated with cumulative low dose exposures in radiology is less certain and there is debate as to whether the risk is linear or if there is a practical threshold below which there is no increased cancer risk [17]. Given this uncertainty, it is a difficult area for both patients and clinicians to navigate and specialist and subspecialist guidance and education from radiologists is required.

Headline reporting in the general media and medical literature regarding radiation exposure can generate fear and misunderstanding amongst patients resulting in anxiety which negatively impacts on quality of life, as well as avoidance of necessary imaging tests [19, 31, 32]. Patients are generally aware that radiation exposure can be associated with cancer, but there is a lack of understanding about the nature and magnitude of this risk and how it applies to different types of examination. Most patients (85–88%) underestimate the risk associated with CT when compared to x-rays [33, 34]. In one study, 34% of patients attending for outpatient CT studies did not realise the scan exposed them to radiation and only 3% considered radiation when thinking about the examination [34]. In a survey of the general public, 58% were unaware CT scans involved radiation [35]. Interestingly, patients who accessed health information from the internet are more likely to have concerns about radiation exposure [33].

Patient-centred care requires medical professionals and patients to understand and discuss the risks and benefits of radiology examinations in a balanced manner to allow patients to come to an informed decision [31, 36]. Patients will often ask health professionals about the radiation dose and resultant risks associated with a particular examination [37]. Health literacy influences patients’ knowledge of radiology procedures and radiation exposure, and their acceptance of imaging tests [37, 38]. In this context, it is concerning that in addition to the misunderstanding amongst patients, current and future clinicians also often lack sufficient knowledge regarding radiation safety [18, 39]. Radiologists may, therefore, play a key role within the patient-centred care model through the provision of widely available online materials detailing reliable and accurate specialist knowledge on radiation safety. If these online PEMs are written at an appropriate level, clinicians may refer patients to them when a particular imaging test is being considered. They may also serve as a reliable source of information for clinicians themselves.

As demonstrated in this study, there has been a failure to make substantial progress in improving the readability of online PEMs. Various contributing factors have been proposed such as the high level of education of the authors, the complexity of modern medical practice, and a determination not to provide incomplete information for legal reasons [25]. There is ever-increasing awareness of the problem across all medical specialities, and several guidelines are available on producing education materials of suitable readability [7, 8, 16]. Other initiatives include an online tool created by the CDC to help develop content with clear communication [40]. In the USA, a 2010 health literacy action plan provides a framework for organisations to improve the readability of information they disseminate and promotes an evidence-based approach to improving health literacy practices [11].

With regards to radiation safety, most patients report an insufficient understanding of medical and scientific terms used by physicians to comprehend the information which is provided [35]. In addition, there is a preference for explanations of radiation risk which are based on the equivalent length of exposure to background radiation or number of chest radiographs [34]. Specific factors such as these should also be considered in addition to general guidelines and frameworks when creating online PEMs in radiology. It may be most appropriate for personnel trained in medical writing to help rewrite education material for patients in simpler terms while retaining detail and accuracy [41].

Advancing health literacy by improving the readability of online PEMs may benefit patients and clinicians. Patients who are well informed on the risks and benefits of an intervention are less likely to request unnecessary tests, helping clinicians avoid the costly overuse of unwarranted medical imaging—though final responsibility lies with the doctor [42]. For radiologists, online PEMs are a means to improve communication with patients and provide reliable information from a trusted source. Improved health literacy can reduce uncertainty and anxiety for patients regarding radiation exposure and facilitate informed decision-making. Currently, the public report that insufficient information is provided regarding the risks of radiation exposure [35].

Our study has several limitations. The readability formulae used are not validated in analysing healthcare literature but are commonly used in education and consistent with methods of analysis in previous studies. Multiple measures that emphasise various aspects of readability were used to enhance our validity. As each formula determines the difficulty level of a passage of text based on the number of letters per word, syllables per word or words per sentence, words with few syllables such as “sievert” may generate a low readability level despite being unfamiliar to patients. Conversely, the multisyllabic structure of many medical terms can cause a small artificial increase in reading level scores. In addition, readability formulae only consider the written information in articles and not visual and non-textual materials which can enhance comprehension such as figures, tables, and multimedia.

## Conclusion

This study demonstrates that the readability of online PEMs related to radiation safety is still written at much higher than recommended reading levels. Radiation safety is a topic in which the specialist training of radiologists is crucial to provide guidance and education to patients on the risks and benefits. Improving the readability of online PEMs relating to radiation safety is a key initiative to support clinicians and to encourage patients to use reliable information from established medical sources as the basis for healthcare decisions. While there are encouraging signs and a commitment from resources such as RadiologyInfo.org to improve the readability of online PEMs, this has not yet translated into a substantial overall improvement. Ongoing analysis of the readability of online PEMs in the coming years is required to support and inform the improvement process. For radiologists, involvement in the creation of online PEMs is an important method to increase communication with patients, support colleagues in other medical specialities, and play a part in the move to patient-centred value-based care.

## Supplementary Information

**Additional file 1. Appendix 1**. Full List of ‘Wordy’ Items With Suggested Alternatives Produced by Readability Studio Software. **Appendix 2.** Titles of online articles analysed

## Data Availability

Not applicable.

## Abbreviations

PEM: Patient education material
RGL: Reading grade level
