# Supplementary material for: Readability of patient education materials related to radiation safety: What are the implications for patient-centred radiology care?
Source: Insights Imaging. 2021 Oct 21;12:148. doi: 10.1186/s13244-021-01094-3 (PMC8531160; doi:10.1186/s13244-021-01094-3)
Supplement: Supplementary file 1 — Additional file 1. Appendix 1. Full List of ‘Wordy’ Items With Suggested Alternatives Produced by Readability Studio Software. Appendix 2. Titles of online articles analysed [file 13244_2021_1094_MOESM1_ESM.docx]

**ELECTRONIC SUPPLEMENTARY MATERIAL**

**Appendix 1.** Full List of ‘Wordy’ Items With Suggested Alternatives Produced by Readability Studio Software

| Wordy Item | Suggested Alternative |
| --- | --- |
| abbreviated | shortened |
| abdomen | belly, stomach |
| ability | skill |
| abolished | got rid of, did away with |
| absolutely | wholly |
| abundant | enough |
| academy | school |
| acceptable | welcome |
| accessible | easy to reach |
| accommodate | adjust, adapt, fit |
| accompany | go with |
| accomplish | do, carry out |
| accomplished | did, done |
| accordingly | so, just so |
| accumulate | collect |
| accuracy | correctness, exactness |
| accurate | correct, exact |
| achievable | doable, makeable |
| achieve | do, make |
| acknowledging | admitting, expressing thanks for |
| acquire | gain, get |
| actual | real |
| actually | really |
| adapted | made fit |
| additional | added, extra |
| adequate | enough |
| adhere | stick to, follow |
| adjacent | next to |
| adjacent to | close to, near, next to, beside, by |
| adjustment | settlement |
| administer | give |
| advantage | plus |
| adversary | enemy |
| adverse | harmful |
| advise | tell, recommend |
| aggregate | collect |
| alleviated | made easier |
| alternate | take turns (between), every other (adj.) |
| alternative | choice |
| an alternative | any other, another |
| analysis | review, breakdown, exam, study |
| annually | yearly |
| anomalously | abnormally |
| anticipated | expected, awaited |
| anxieties | fears |
| apparent | clear, plain |
| appear | seem, come |
| appropriate | proper (adj.), set aside (verb) |
| approval | praise, consent |
| approximately | about |
| are applicable to | apply to |
| are comparable to | compare to |
| are concerned about | worry, fear |
| are dependent on | depend on, hinge on |
| as a consequence of | because, because of, from, since |
| as a general rule | generally, in general, customarily |
| as opposed to | compared to |
| as well as | and, also |
| aspirations | longings, dreams, goals |
| assembled | gathered |
| assembly | meeting |
| assist | aid, help |
| assistance | help |
| at all times | always |
| at present | now, today |
| attempt | try |
| attractive | pleasing |
| authorize | give power |
| available | offered, ready |
| bear in mind | consider, note |
| beneficial | helpful |
| beneficiary | person helped |
| benefit | help |
| bilateral | two-sided |
| bona fide | in good faith |
| by virtue of | by, because of, under |
| cannot be avoided | must, should |
| capability | ability |
| capable | able |
| capacity | ability, power, position |
| category | class, group |
| certainly | surely |
| characteristic | trait |
| characterize | describe |
| coalition | union, joining |
| collaborates | works together |
| collection | mass, heap |
| combines | joins |
| commitments | pledges |
| compensate | pay |
| comply with | follow, obey |
| component | part |
| composed | made up, created, calm (adj.) |
| composition | make-up |
| comprises | forms, includes |
| conception | thought, idea, creation |
| concerning | about, on |
| concludes | closes, ends |
| conclusion | close, end |
| conclusive | final |
| congenital | inborn |
| conscious | aware |
| consequences | results |
| consequently | so |
| considerable | much |
| constantly | always |
| construct | build |
| consumes | uses up |
| contain | have, hold |
| contaminated | tainted |
| continue | keep, keep on |
| contribute | give, help |
| convenient | handy |
| conversion | change |
| cooperate | help |
| cooperative | helpful |
| course of action | course, direction, plan |
| create | make |
| criteria | requirements |
| customary | usual, accepted |
| deem | think |
| deliberately | on purpose |
| demonstrate | show |
| dense | thick |
| density | thickness |
| depleted | emptied, empty (adj.) |
| designate | appoint, choose, assign |
| desire | wish |
| determine | decide, figure |
| detrimental | harmful |
| develop | make, grow |
| deviate | stray, turn away |
| difficult | hard |
| difficulty | trouble |
| discover | find out |
| disintegrate | break up |
| disintegrates | breaks up |
| dispatch | send |
| dispersed | scattered |
| disrupt | interrupt, confuse |
| disseminate | issue, send out |
| distinguish | tell apart |
| duplicate | copy |
| economical | thrifty |
| educational institutions | schools, colleges, universities |
| elevate | raise, lift up |
| elevation | height |
| eliminate | cut, drop |
| emanating | coming from |
| emphasize | stress |
| employment | work, job, use |
| encounters | meets |
| encourage | urge |
| enormous | large, huge |
| ensure | make sure |
| equivalent | equal |
| establish | set up, prove |
| evaluate | check, rate |
| examination | check |
| examine | check, look at |
| exchange | trade |
| execution | completion |
| expense | cost, fee |
| explain | show, tell |
| explicit | clear |
| exterior | outside |
| external | outer |
| fabricate | build, make |
| facilitate | ease, help |
| familiar | known |
| feasible | can be done |
| final | last |
| for that reason | therefore, thus |
| for the purpose of | to, for, of |
| frequently | often |
| function | act, role |
| fundamental | basic |
| general | broad |
| general public | public |
| generally | broadly |
| generate | create, make |
| has an impact on | affects, influences |
| have a tendency | tend to |
| have the ability to | can |
| having an impact on | affecting, influencing |
| hazardous | risky, unsafe |
| hesitate | pause |
| heterogeneous | varied |
| horizontally | sideways |
| however | but |
| identical | same |
| identification | ID |
| identify | name, find |
| illustrate | draw, show |
| immediately | at once, right away, right now |
| imminent | near |
| impact | hit, change |
| implement | carry out, do |
| in a timely manner | at once, on time, quickly, swiftly |
| in certain cases | at times, sometimes |
| in comparison to | compared to |
| in excess of | more than |
| in many cases | mostly, most of these, often, usually |
| in most cases | mostly, most of these, often, usually |
| in operation | operative, active, working |
| in order to | to, for |
| in reference to | about, on, for, as for, in, of, over, respecting, to, toward, with |
| in relation to | about, for, on; about, in, on, to, toward, with |
| in some cases | at times, sometimes |
| in some situations | at times, sometimes |
| in terms of | as for |
| in the amount of | for, of |
| in the case of | in, with, if, by, for (or delete) |
| in the context of | in, about, for, of |
| in the course of | during, throughout |
| in the event of | if, should |
| in the middle of | amid, in between |
| in the presence of | with, before |
| in the process of | while |
| in the sense that | in that |
| in the vicinity of | close to, near, about, close by, in, nearby, around, round, close |
| inadvertent | careless |
| incision | cut |
| incorporate | blend, join, mix |
| incorporate into | add, include |
| indicate | show |
| indication | clue, sign |
| individual | person, single |
| individuals | people |
| individuals who | those who |
| inexhaustible | tireless |
| infrastructure | system |
| infrequent | rare |
| initial | first |
| initially | at first |
| initiate | begin |
| initiated | started |
| institution | office, company, school |
| insufficient | not enough |
| internal | inner, inside |
| invaluable | priceless |
| investigate | review, check, look over |
| is composed of | comprises |
| is concerned with | worries, fears |
| justified | proved/proven |
| legitimate | valid, real, proper, normal |
| limitation | limit |
| limited number of | a few, little, meager, not many, scant, only so many, some, spare, sparse |
| locate | find |
| location | place |
| luminous | bright |
| magnitude | size |
| maintain | keep, support |
| majority | most |
| make a determination | determine |
| malformation | abnormal shape, deformity |
| manually | by hand |
| manufactured | made |
| massive | large |
| maximum | most, greatest |
| may or may not | may, may not |
| minimise | decrease, lessen |
| mitigate | moderate |
| modify | change |
| monitor | check, watch |
| multiple | many |
| necessary | needed |
| necessitated | caused, needed |
| neighboring | next to |
| neutralize | offset |
| no matter how | however |
| notification | alert, notice, message, statement, warning |
| notify | let know, tell |
| numerous | many |
| objective | aim, goal |
| obligation | duty, pledge |
| obscured | hidden |
| observe | see |
| obstruction | block, hurdle |
| obtain | get |
| occurrence | event |
| on the surface | seemingly, apparently |
| operate | run, work |
| opportunity | chance |
| optimum | best, greatest |
| option | choice, way |
| organizations | companies |
| outside of | outside (unless proceeding a pronoun) |
| participating | taking part |
| particular | specific |
| perform | do |
| permit | let |
| personnel | staff, people |
| pertaining to | about, regarding |
| phantoms | ghosts |
| physician | doctor |
| plan of action | plan, strategy |
| portion | part |
| position | place |
| possess | have, own |
| precipitation | rain, snow, dew, frost |
| predominantly | superiorly |
| preparation | readiness |
| preparedness | readiness |
| previous | earlier, past |
| primary | main, first |
| prior to | before |
| probability | chance |
| proceed | do, go on |
| produced | made |
| program | plan |
| purchase | buy |
| pursuant to | under, by, following |
| radiant | bright |
| rapid | quick |
| recommend | suggest |
| regulation | rule, law |
| reinforced | strengthened |
| relates to | about, as for, for, in, of, on, over, to, toward, with |
| relinquishes | gives up |
| remain | stay |
| remiss | slack, negligent |
| request | ask |
| require | need |
| requisite | needed, necessity |
| residence | house, home |
| responsibility | duty, job |
| result in | lead to |
| retain | keep, hold |
| review | check |
| rigorous | harsh, strict |
| similar | like |
| simplify | ease |
| simultaneously | at the same time |
| strategies | plans |
| stringent | strict, tight |
| submit | send, give |
| subsequent | later, next |
| substantial | real, strong, large |
| sufficient | enough, ample |
| supplemental | added, extra |
| terminate | end, stop |
| therefore | so, thus |
| thereof | its, their |
| through the use of | by, with |
| to some degree | in a sense, somewhat, partly |
| total number of | total |
| trace amount | trace |
| transmits | sends |
| typically | often |
| uncertainty | doubt |
| use up | use |
| usually | often |
| utility | tool |
| utilize | use |
| validated | confirmed |
| validity | truth |
| value | cost, worth |
| variation | change, difference |
| vertically | upward |
| via | in, on, by |
| visualize | picture |
| whereas | since |

**Appendix 2.** Titles of online articles analysed

**Patient Info**

1. CT scan
2. Mammogram
3. X-ray test

**RadiologyInfo.org**

1. Are screening exams worthwhile?
2. Can medical diagnostic imaging procedures cause future reproductive problems?
3. Can the exams hurt the baby of a pregnant woman?
4. Children and Radiation Safety
5. Computer Tomography (CT) Safety During Pregnancy
6. Does radiation risk depend on age?
7. Does radiation risk depend on gender?
8. Fetal and Gonadal Shielding
9. How big is the risk from medical imaging to future generations?
10. How much dose do I get from different imaging procedures?
11. How much radiation is too much?
12. Is diagnostic medical radiation safe?
13. I've had many CT scans. Should I be concerned?
14. Radiation Dose in X-Ray and CT Exams
15. Safety in X-ray, Interventional Radiology and Nuclear Medicine Procedures
16. What are the benefits of CT scans?
17. What is radiation dose?
18. What is the radiology community doing to appropriately manage radiation exposure?

**Centers for Disease Control and Prevention**

1. ALARA – As Low As Reasonably Achievable
2. Health Effects of Radiation: Health Effects Depend on the Dose
3. Nuclear Medicine Procedures
4. Radiation in Medicine: CT Scans
5. Radiation in Medicine – Fluoroscopy
6. Radiation in Medicine – Medical Imaging Procedures
7. Radiation in Medicine: X-rays

**United States Environmental Protection Agency**

1. Protecting Yourself from Radiation
2. Radiation Basics
3. Radiation Health Effects
4. Radiation Sources and Doses
5. Radiation Terms and Units
6. Radionuclides

**International Atomic Energy Agency**

1. X-Rays – What Patients Need to Know
2. Radiation – What Patients Need to Know
3. Computed Tomography (CT) – What Patients Need to Know
4. Interventional Procedures – What Patients Need to Know
5. Pregnancy and Radiation – What Patients Need to Know
6. Children and Radiation – What Patients Need to Know

**U.S. Food and Drug Administration**

1. Computed Tomography (CT)
2. Dental Cone-Beam Computed Tomography
3. Dose Matters: FDA's Guidance on Children's X-rays
4. Fluoroscopy
5. Full-Body CT Scans - What You Need to Know
6. Mammography
7. Medical X-Ray Imaging
8. Paediatric X-Ray Imaging
9. Radiography
10. Reducing Radiation from Medical X-rays
11. Whole-Body CT Screening--Should I or shouldn't I get one?
12. X-rays, Pregnancy and You

**Health Physics Society**

1. Common Sources of Radiation
2. Radiation and Risk
3. Radiation Exposure and Pregnancy
4. Radiation Exposure From Medical Exams and Procedures

**Image Gently**

1. What Parents Should Know about CT Scans for Children
2. What Parents Should Know About Medical Radiation Safety in Pediatric Interventional Radiology
3. What Parents Should Know about Medical Radiation Safety
4. X-Rays for Children: What Parents Should Know About Radiation Protection in Medical Imaging
5. What You Should Know About Pediatric Nuclear Medicine and Radiation Safety
6. Fluoroscopy – Parents

**InsideRadiology**

1. Computed Tomography (CT)
2. Plain Radiograph/X-ray
3. Radiation Risk of Medical Imaging for Adults and Children

**Mayo Clinic**

1. Chest X-Rays
2. CT Scan
3. CT scans: Are they safe?
4. X-ray

**MedlinePlus**

1. Radiation Exposure
2. Radiation Sickness
3. X-Rays

**Nuclear Regulatory Commission**

1. Detecting Radiation
2. Radiation Basics
3. Glossary
4. High Radiation Doses
5. Measuring Radiation
6. Information for Radiation Workers
7. Radiation and Its Health Effects
8. Radiation Exposure and Cancer

**Society for Paediatric Radiology**

1. CT Scan Information for Parents
2. Digital radiography
3. Nuclear Medicine Studies Information for Parents

**European Society of Radiology**

1. I had an X-ray but didn’t know that I’m pregnant. And now?
2. Risk of radiation induced cancer and exposure to low dose computed tomography for lung cancer screening
3. My child will have a CT scan! A guide for parents
4. What Patients Should Know: Radiation Dose in Computed Tomography
5. Interventional procedures and radiation dose - What the patient should know
6. What Patients Should Know – Role of the CT Team
